# Clinico-Pathological Outcomes of Patients With Crescentic Glomerulonephritis: A Single-Center Study

**DOI:** 10.7759/cureus.38777

**Published:** 2023-05-09

**Authors:** Abdullah Z Alsuheili, Hanadi Alhozali, Ayar A Bukhari, Mohammad A Khan, Abdulaziz S Alzahrani, Suhail K Abualnaja, Reem A Al Zahrani

**Affiliations:** 1 Faculty of Medicine, King Abdulaziz University Hospital, Jeddah, SAU; 2 Faculty of Medicine, Department of Medicine, Nephrology Unit, King Abdulaziz University Hospital, Jeddah, SAU; 3 Pathology, King Abdulaziz University Hospital, Jeddah, SAU

**Keywords:** interstitial fibrosis and tubular atrophy, ifta, rpgn, rapidly progressive glomerulonephritis, crgn, crescentic glomerulonephritis

## Abstract

Background

Crescentic glomerulonephritis (CrGN) is a pathological description of rapidly progressive glomerulonephritis (RPGN). It is characterized by renal failure and is associated with a grave prognosis. This study aimed to investigate the clinical outcomes of patients diagnosed with crescentic glomerulonephritis at the King Abdulaziz University Hospital (KAUH) in Jeddah, Saudi Arabia.

Method

This retrospective study included patients with CrGN who underwent treatment at the nephrology department at KAUH from June 2021 to August 2022. We collected and analyzed data from 56 patients diagnosed with CrGN on the basis of renal biopsies between 2002 and 2015.

Result

The study included 17 cases of CrGN. The mean age of patients at the time of diagnosis was 18.06 ± 13.49 years. The distribution of histological findings showed that cellular crescents (94.1%) and interstitial fibrosis and tubular atrophy (IFTA) (76.5%) were the most commonly observed histological findings. The most common underlying etiology was lupus nephritis (41.2%). Regarding the lab results, the mean serum creatinine level at admission was 378.88 ± 273.27 μmol/L, proteinuria was 1.53 ± 1.23 and glomerular filtration rate (GFR) level was 36.94 ± 45.08 mL/min. The factors associated with poor renal outcome were IFTA (P=0.01), phosphate level before discharge, serum creatinine level before and after discharge (P=0.032), and GFR level after discharge (P=0.001).

Conclusion

Crescentic glomerulonephritis is an important cause of acute kidney injury due to its potential to result in severe glomerular injury. In our study, 12 out of 17 patients experienced poor renal outcomes, which were associated with a high risk of morbidity and mortality. Therefore, early detection and treatment of CrGN is crucial in order to manage the disease.

## Introduction

The glomerulonephritides (GN) are a group of disorders characterized by severe inflammation and glomerular damage. Glomerulonephritis can be further classified on the basis of histological characteristics and clinical presentation [1]. Nephritic syndrome, also known as rapidly progressive glomerulonephritis (RPGN), has a clinical presentation characterized by edema, renal failure, active urinary sediments, and proteinuria. It is caused by multiple diseases that result in the formation of crescents observed on renal biopsy; hence, it is also known by the pathological term crescentic glomerulonephritis (CrGN) [2]. RPGN necessitates prompt treatment in order to avoid progressive renal failure or death [2,3]. CrGN can be classified into three disease categories based on the immunofluorescence microscopic pattern observed on kidney biopsy: linear, granular, and pauci-immune. A linear pattern implies anti-glomerular basement disease, while granular staining is present in immune-complex mediated diseases, such as lupus nephritis and post-infectious glomerulonephritis. Most cases of glomerulonephritis with a paucity-immune pattern are induced by antineutrophil cytoplasmic autoantibody (ANCA)-associated vasculitis [4]. Crescentic glomerulonephritis is diagnosed on the basis of laboratory findings, and a kidney biopsy that reveals a crescent-shaped development in the renal capsule (Bowman's capsule) [3]. However, CrGN is uncommon. In a study conducted in Italy in 1997, only 13% of 13,835 patients who underwent kidney biopsy exhibited CrGN [5]. In 2018, a study conducted in Taiwan demonstrated that 2.6% of 1281 individuals who underwent renal biopsy developed CrGN [6], while a study conducted in Turkey in 2020 discovered that only 5.2% of 3875 patients who underwent renal biopsy developed CrGN [7]. A local study conducted in 2010 found a 3.2% incidence of CrGN based on 233 renal biopsies obtained from patients aged 17-43 years [8]. CrGN is a concerning clinical entity characterized by ethnic disparities in disease genesis, severity, and clinical prognosis.

Despite its rarity, the diagnosis of CrGN is considered an acute emergency in the field of nephrology and requires prompt diagnosis and management [9]. However, in Saudi Arabia, particularly in the western region, there is a lack of data regarding the renal outcomes of patients diagnosed with crescentic glomerulonephritis. Therefore, this study aimed to investigate the clinicopathological outcomes of crescentic glomerulonephritis in patients at King Abdulaziz University Hospital (KAUH) in Jeddah, Saudi Arabia.

## Materials and methods

Study design, setting and time frame

This retrospective study was conducted at KAUH from June 2021 to August 2022. We collected and analyzed data from 56 patients diagnosed with biopsy-proven CrGN between 2002 and 2015. Seventeen patients met the inclusion criteria. CrGN was defined as the presence of proliferation of parietal cells forming two or more cell layers filling Bowman’s space in more than 50% of glomeruli in the renal biopsy [10]. All patients diagnosed with crescentic glomerulonephritis between 2002 and 2015 were included. Patients with incomplete data were excluded.

Data collection

A pre-designed checklist was used to collect data about patients’ demographics, body mass index BMI, admission year, histopathological results, etiology of CrGN, and proteinuria level at admission, before discharge and two months after discharge. Data such as laboratory test results at admission, before discharge and two months after discharge and patient outcomes were also collected. The outcomes studied included complete and partial renal recovery, chronic kidney disease (CKD), end-stage renal disease (ESRD), and mortality at the end of two month follow-up period. Complete recovery was defined as normal urinalysis and estimated glomerular filtration rate (eGFR) > 90 mL/min/1.73 m2. Partial recovery was defined as eGFR > 60 mL/min/1.73 m2 with the presence of abnormal urinalysis: microscopic hematuria, 1+ proteinuria with or without red blood cell (RBC) casts. CKD was defined as an eGFR > 60 mL/min/1.73 m2. ESRD was defined as an eGFR > 15 mL/min/1.73 m2.

Ethical considerations

Ethical approval for the study was obtained from the Research Ethics Committee of King Abdulaziz University, Jeddah, Saudi Arabia (Reference no. 106-21).

Data analysis

Statistical Package for Social Sciences (SPSS) version 26 (IBM Corp., Armonk, NY, USA) was used to analyze the data. The Chi-squared test (χ2) was used to assess the association between qualitative data reported as numbers and percentages. Quantitative data was presented as mean and standard deviation (Mean ± SD) and Kruskal Wallis test and one way ANOVA test were used according to the data normality. A p-value of less than 0.05 was considered statistically significant.

## Results

Medical records of a total of 17 patients with CrGN were reviewed. Eleven (64.7%) patients were male and six (35.3%) were female. Of the 17 patients, nine (52.9%) were Saudi nationals and eight (47.1%) were of different nationalities. The mean age of the patients at the time of diagnosis was 18.06 ± 13.49 years. The mean BMI was 18.06 ± 11.15 kg (Table 1).

**Table 1:** Distribution of patients according to their demographics, BMI, and admission year (total number of patients: 17) BMI: Body mass index

Variable	No. (%)
Age at diagnosis	18.06 ± 13.49 years
BMI	18.06 ± 11.15
Gender	
Female	6 (35.3)
Male	11 (64.7)
Nationality	
Saudi	9 (52.9)
Non-Saudi	8 (47.1)
Year of admission	
2002	2 (11.8)
2009	2 (11.8)
2010	2 (11.8)
2011	3 (17.6)
2012	1 (5.9)
2013	1 (5.9)
2014	5 (29.4)
2015	1 (5.9)

Histopathological findings are demonstrated in Table 2. The distribution of histological findings showed that cellular crescents (94.1%) and interstitial fibrosis and tubular atrophy (IFTA) (76.5%) were the most common histological findings observed. The common etiologies included granular CrGN (82.3%) with lupus nephritis predominance (41.2%), linear CrGN (11.8%), and pauci-immune CrGN (5.9%) (Table 2).

**Table 2:** Distribution of patients according to histopathological findings, etiology of CrGN, microscopic and macroscopic hematuria at admission, before discharge CrGN: Crescentic glomerulonephritis GBM: Glomerular basement membrane

Variable	No. (%)
Histopathology results	
Cellular crescents	16 (94.1)
Fibrocellular crescents	8 (47.1)
Fibrous crescent	11 (64.7)
Interstitial fibrosis and tubular atrophy (IFTA)	13 (76.5)
Glomerular hypercellularity	3 (17.6)
Mesangial cell proliferation	9 (52.9)
Endocapillary proliferation	6 (35.3)
Neutrophilic infiltration	6 (35.3)
Vasculopathy	2 (11.8)
Fibrinoid necrosis and Karryorrhectic cells	6 (35.3)
Global sclerosis	10 (58.8)
GBM thickness	3 (17.6)
Tamm-Horseful protein cast with focal calcification	1 (5.9)
Segmental melangiolysis	1 (5.9)
Ischemic wrinkling of the glomerular basement membrane.	1 (5.9)
hyaline arteriolosclerosis	1 (5.9)
Etiology of CrGN	
Goodpasture syndrome	2 (11.8)
IgA vasculitis	1 (5.9)
Lupus nephritis	7 (41.2)
Membranoproliferative nephropathy	2 (11.8)
Pacui-immune GN(Wegener)	1 (5.9)
Post-infectious glomerulonephritis	4 (23.5)
Microscopic hematuria at admission	
No	11 (64.7)
Yes	6 (35.3)
Macroscopic hematuria at admission	
No	11 (64.7)
Yes	6 (35.3)
Microscopic hematuria before discharge	
No	8 (47.1)
Yes	9 (52.9)
Macroscopic hematuria before discharge	
No	17 (100)
Yes	0 (0.0)

According to laboratory findings, the mean serum creatinine level at admission was 378.88 ± 273.27 μmol/L, proteinuria 1.53 ± 1.23 and GFR level was 36.94 ± 45.08 mL/min. Before discharge, the mean serum creatinine was 327.18 ± 318.88 μmol/L, proteinuria 2.5 ± 1.08 and GFR level was 62.65 ± 68.25 mL/min. Two months after discharge, the serum creatinine was 399.14 ± 46.29 μmol/L, proteinuria 1.19 ± 0.63 and GFR level was 65.79 ± 58.12 mL/min. The results of other laboratory tests at admission, before discharge, and two months after discharge are reported in Table 3.

**Table 3:** Laboratory test results at admission, before discharge and two months after discharge BUN: Blood urea nitrogen GFR: Glomerular filtration rate

Variable	No. (%)
Laboratory tests at admission	
Sodium (mmol/L)	135.71 ± 4.87
Potassium (mmol/L)	4.79 ± 1.12
Calcium (mmol/L)	1.96 ± 0.25
Phosphate (mmol/L)	1.83 ± 0.27
BUN (mmol/L)	25.59 ± 15.69
Creatinine (μmol/L)	378.88 ± 273.27
Proteinuria	1.53 ± 1.23
GFR	36.94 ± 45.08
Laboratory tests before discharge	
Sodium (mmol/L)	138.65 ± 4.78
Potassium (mmol/L)	3.96 ± 1.29
Calcium (mmol/L)	1.33 ± 1.02
Phosphate (mmol/L)	1.09 ± 1.08
BUN (mmol/L)	20.12 ± 12.67
Creatinine (μmol/L)	327.18 ± 318.88
Proteinuria	2.5 ± 1.08
GFR	62.65 ± 68.25
Laboratory tests after 2 months of discharge	
Creatinine (μmol/L)	399.14 ± 46.29
Proteinuria	1.19 ± 0.63
GFR	65.79 ± 58.12

**Figure 1:**
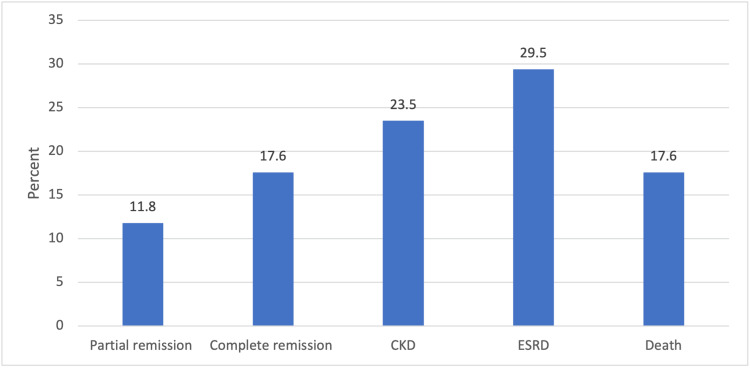
Percentage distribution of patients according to their outcomes CKD: Chronic kidney disease ESRD: End stage renal disease

Furthermore, the patients were categorized into two groups based on their prognosis: the first group included patients with good prognosis (complete and partial remission), and the second group included patients with poor prognosis (CKD, ESRD, and death). The majority of the cases (12, 70.5%) demonstrated poor prognosis, while five (29.4%) cases had a good prognosis (Figure 1).

No significant relationship was observed between the outcomes and patient demographics, BMI, duration of hospital stay, and etiology (P ≥ 0.05) (Table 4).

**Table 4:** Relationship between outcomes and patient demographics, BMI, duration of hospital stay, and etiology of CrGN * = Kruskal Wallis test.  ** = One Way ANOVA test CKD: Chronic kidney disease ESRD: End stage renal disease BMI: Body mass index CrGN: Crescentic glomerulonephritis

	Outcome					χ^2^	p-value
Variable	Partial remission	Complete remission	CKD	ESRD	Death		
Age at diagnosis	13 ± 0.001 years	9 ± 1.15 years	23.75 ± 15.06 years	15.8 ± 10.47 years	26.33 ± 24 years	4*	0.351
BMI	27.78 ± 11	11.56 ± 10.02	26.43 ± 4.88	16.9 ± 11.33	7.85 ± 11.1	4**	0.112
Gender							
Female	1 (16.7)	0 (0.0)	1 (16.7)	2 (33.3)	2 (33.3)	3.35	0.501
Male	1 (9.1)	3 (27.3)	3 (23.3)	3 (27.3)	1 (9.1)		
Duration of hospital stay (days)	23.5 ± 20.5 days	15.66 ± 4.04 days	25 ± 16.26 days	76.8 ± 38.08 days	36.66 ± 12.6 days	4*	0.06
Etiology of CrGN						23.84	0.249
Goodpasture syndrome	0 (0.0)	0 (0.0)	0 (0.0)	1 (50)	1 (50)		
IgA vasculitis	0 (0.0)	0 (0.0)	1 (100)	0 (0.0)	0 (0.0)		
Lupus nephritis	1 (14.3)	0 (0.0)	2 (28.6)	3 (42.9)	1 (14.3)		
Membranoprolefative nephropathy	0 (0.0)	0 (0.0)	1 (50)	0 (0.0)	1 (50)		
Pacui-immune GN( wegners)	0 (0.0)	0 (0.0)	0 (0.0)	1 (100)	0 (0.0)		
Post-infectious glomerulonephritis	1 (25)	3 (75)	0 (0.0)	0 (0.0)	0 (0.0)		

There were significant differences between the five outcomes observed in IFTA (P=0.01). This was found to be significantly less in the good prognosis group (partial remission, 15.7%) as partial remission (15.4%) and no patients with complete remission had it, compared with the poor prognosis group (84.7%) (Table 5).

**Table 5:** Relationship between outcomes and patients’ histopathological results GBM: Glomerular basement membrane

Variable	Outcome					χ^2^	p-value
	Partial remission	Complete remission	CKD	ESRD	Death		
Cellular crescents	2 (12.5)	3 (18.8)	4 (25)	4 (25)	3 (18.8)	2.55	0.636
Fibrocellular crescents	2 (25)	1 (12.5)	3 (37.5)	1 (12.5)	1 (12.5)	5.42	0.246
Fibrous crescent	2 (18.2)	1 (9.1)	4 (36.4)	3 (27.3)	1 (9.1)	5.9	0.206
Interstitial fibrosis and tubular atrophy (IFTA)	2 (15.4)	0 (0.0)	4 (30.8)	5 (38.5)	2 (15.4)	13.29	0.01
Glomerular hypercellularity	1 (33.3)	1 (33.3)	0 (0.0)	0 (0.0)	1 (33.3)	4.38	0.356
Mesangial cell proliferation	2 (22.2)	2 (22.2)	3 (33.3)	0 (0.0)	2 (22.2)	8.63	0.071
Endocapillary proliferation	2 (33.3)	2 (33.3)	1 (16.7)	1 (16.7)	0 (0.0)	7.29	0.121
Neutrophilic infiltration	1 (16.7)	2 (33.3)	1 (16.7)	1 (16.7)	1 (16.7)	2.18	0.702
Vasculopathy	0 (0.0)	0 (0.0)	1 (50)	1 (50)	0 (0.0)	2.06	0.723
Fibrinoid necrosis and Karryorrhectic cells	2 (33.3)	2 (33.3)	0 (0.0)	1 (16.7)	1 (16.7)	7.65	0.105
Global sclerosis	2 (20)	1 (10)	3 (30)	3 (30)	1 (10)	3.44	0.486
GBM thickness	0 (0.0)	0 (0.0)	0 (0.0)	3 (100)	0 (0.0)	8.74	0.068
Tamm-Horseful protein cast with focal calcification	0 (0.0)	0 (0.0)	0 (0.0)	1 (100)	0 (0.0)	2.55	0.636
Segmental melangiolysis	0 (0.0)	0 (0.0)	0 (0.0)	1 (100)	0 (0.0)	2.55	0.636
Ischemic wrinkling of the glomerular basement membrane.	0 (0.0)	0 (0.0)	0 (0.0)	1 (100)	0 (0.0)	2.55	0.636
Hyaline arteriolosclerosis	0 (0.0)	0 (0.0)	0 (0.0)	1 (100)	0 (0.0)	2.55	0.636

A significant relationship was observed between the outcomes and serum phosphate and creatinine levels before discharge. Patients with good outcomes had lower phosphate and creatinine levels as compared with those with poor outcomes. The mean phosphate value was: CKD 0.35 ± 0.7, ESRD 2.07 ± 0.28 mmol/L and death 1.57 ± 1.29 mmol/L compared with complete remission 0.3 ± 0.51 mmol/L and partial remission 0.62 ± 0.88 mmol/L (P=0.045). The mean creatinine level was CKD (stages 1-4) 166.75 ± 38.89 μmol/L, ESRD 668.6 ± 289.7 μmol/L and death 396.6 ± 357 μmol/L compared with 46.67 ± 11.24 μmol/L complete remission and partial remission 111 ± 2.82 μmol/L (P=0.032). Two months after discharge, significant trends were observed in creatinine level and GFR level. Patients with good outcomes had lower levels of creatinine. Patients in complete remission had mean creatinine of 36.67 ± 7.37 μmol/L and those in partial remission had 73.5 ± 19.09 μmol/L compared with patients with poor outcomes that had high levels of creatinine. Patients in CKD had mean creatinine of 109.5 ± 27.67 μmol/L, and those in ESRD had 978.6 ± 188.97 μmol/L (P=0.032). Moreover, patients with good outcomes had a normal range GFR level. Patients in complete remission had a GFR of 126.67 ± 38.07 mL/min, while those in partial remission had a GFR of 114.5 ± 74.24 mL/min compared with abnormal GFR levels that were associated with poor outcomes. Patients with CKD had mean GFR levels of 70.75 ± 22.69 mL/min, while those with ESRD had GFR of 5.8 ± 1.09 mL/min (P=0.001) (Table 6).

**Table 6:** Relationship between outcomes and laboratory test results at admission, one week of treatment, before discharge and two months after discharge BUN: Blood urea nitrogen GFR: Glomerular filtration rate

Variable	Outcome					Kruskal Wallis test	p-value
	Partial remission	Complete remission	CKD	ESRD	Death		
Laboratory tests at admission							
Sodium (mmol/L)	133 ± 5.65	138 ± 8.88	138.5 ± 3.31	136 ± 1.87	31 ± 2.64	1.53*	0.254
Potassium (mmol/L)	5.8 ± 0.56	3.83 ± 1	4.67 ± 1.22	5.04 ± 0.56	4.83 ± 1.85	4	0.298
Calcium (mmol/L)	1.76 ± 0.19	1.93 ± 0.02	2.08 ± 0.23	1.9 ± 0.25	2.45 ± 1.96	1.93*	0.198
Phosphate (mmol/L)	1.79 ± 0.03	2.2 ± 0.54	1.85 ± 0.25	1.68 ± 0.07	1.78 ± 0.276	3	0.216
BUN (mmol/L)	34.85 ± 34.83	34.86 ± 21.52	21.07 ± 6.29	23.46 ± 10.62	19.73 ± 8.26	4	0.802
Creatinine (μmol/L)	212 ± 31.11	128.67 ± 5.5	375.5 ± 149.5	503.6 ± 360.53	537 ± 324.42	1.48*	0.266
Proteinuria	2.5 ± 0.7	1.33 ± 1.15	1.25 ± 1.5	1.4 ± 1.34	1.67 ± 1.52	4	0.792
GFR	32 ± 9.89	64.67 ± 14.97	20.75 ± 14.36	47.6 ± 79.97	16.3 ± 16.19	4	0.175
Laboratory tests before discharge							
Sodium (mmol/L)	143.5 ± 0.7	138.33 ± 3.78	137.6 ± 6.58	134.33 ± 6.8	134.3 ± 3.05	1.67*	0.221
Potassium (mmol/L)	3.5 ± 0.84	3.4 ± 0.6	3.57 ± 0.66	3.78 ± 0.97	5.33 ± 0.27	4	0.718
Calcium (mmol/L)	0.9 ± 1.27	0.6 ± 1.03	0.58 ± 1.16	2.1 ± 0.14	2.06 ± 0.05	4	0.084
Phosphate (mmol/L)	0.62 ± 0.88	0.3 ± 0.51	0.35 ± 0.7	2.07 ± 0.28	1.57 ± 1.29	3.38*	0.045
BUN (mmol/L)	11.2 ± 5.09	10.93 ± 5.77	16.5 ± 9.2	28.08 ± 10.93	26.8 ± 20.35	1.66*	0.222
Creatinine (μmol/L)	111 ± 2.82	46.67 ± 11.24	166.75 ± 38.89	668.6 ± 289.7	396.6 ± 357	4	0.032
Proteinuria	1 ± 0.001	2.33 ± 1.52	3 ± 1.41	2.75 ± 0.5	------	0.83*	0.523
GFR	75.5 ± 38.89	138 ± 44.03	45 ± 22.53	12.8 ± 11.6	85.33 ± 131.3	4	0.063
Laboratory tests after 2 months of discharge							
Creatinine (μmol/L)	73.5 ± 19.09	36.67± 7.37	109.5 ± 27.67	978.6± 188.97	------	4	0.032
Proteinuria	1 ± 0.001	0.67 ± 0.57	1.87 ± 0.18	1.5 ± 0.7	------	3	0.157
GFR level	114.5 ± 74.24	126.67± 38.07	70.75 ± 22.69	5.8 ± 1.09	------	11.36	0.001

## Discussion

Though uncommon, rapidly progressing glomerulonephritis (CrGN) is a major cause of renal impairment. Hence, this study aimed to elucidate the histopathological as well as disease patterns of crescentic glomerulonephritis and its clinical outcomes in patients who underwent treatment at KAUH in Jeddah, Saudi Arabia.

Our study findings differed from previously published study in terms of the etiology of crescentic glomerulonephritis. In our study, lupus nephritis (immune-complex CrGN) accounted for 41.2% of the cases. This is similar to the results of three previously published regional Saudi studies [11-13]. In contrast, other Saudi and Indian reports have reported post-infectious glomerulonephritis (immune-complex CrGN) as the most commonly observed etiology [9,14]. Two studies conducted in Asia identified pauci-immune as their primary etiology [15,16]. 70.5% of our patients had poor outcomes (CKD, ESRD, and death). Studies from India and the United Kingdom have reported similar outcomes [9,17]. In contrast, two regional studies have reported better outcomes for patients with CrGN. This could be attributable to the pediatric population of these studies [12,14]. In this study, the mean age at diagnosis was 18.06 ± 13.49 years, which is younger than reported in the other studies from Saudi Arabia [11,13]. Moreover, we did not observe any significant relationship between age and outcomes. A previously published regional study has reported that the mean age of their patients was 13.2 ± 5.6 years, and younger age were associated with a poor outcomes [12]. In another Saudi study, the mean age was 35.6 years. This study also reported adverse outcomes in younger patients [11]. Finally, according to a South Korean study, the mean age of their patients was 61 ± 15.3 years. In contrast to other studies, they reported that older age is associated with worsening renal outcomes, [16] which could be attributed to the advanced age of the patients included in their study. The patient population was predominantly male in our study as well as in other studies [14,18]. In contrast, the patients were predominantly female in two regional studies [12,13]. Additionally, in our study, no significant associations were observed between sex and outcomes, similar to the study by Oudah et al. [11].

Based on the renal biopsy findings, the most common histolgies were cellular crescents (94.1%), followed by IFTA (76.5%). This is similar to recent studies conducted in India and China, where cellular crescents were the most commonly observed histopathological findings [19,20]. In contrast, Tauhidul et al. reported that the majority of crescents were fibrocellular [21]. Interestingly, we observed a significant relationship between IFTA and clinical outcomes, with a higher percentage of patients with a poor outcome demonstrating IFTA on renal biopsies. Oudah et al. observed that patients with global sclerosis had a poor outcome [11]. In our study, IFTA was the only histopathological factor identified to have an association with outcomes. Similarly, Suceena et al. also observed that the presence of moderate-to-severe IFTA was a significant predictor of poor outcomes [22]. In contrast, two studies reported that the percentage of fibrocellular crescents was predictive of an adverse outcome [14,23]. However, another Indian study observed no statistical relationship between the presence of IFTA or fibrocellular crescents and the outcomes [19]. Our study did not demonstrate a noticeable relationship between the outcome and creatinine level at admission. This is similar to the findings of two previous Asian studies [10,12]. In contrast, different studies have reported that high serum creatinine level at admission is associated with poor outcomes [14,19,24,25]. However, we observed a significant relationship between outcomes and phosphate and creatinine readings before discharge. Those with better outcomes had lower phosphate and creatinine levels. No previous studies have investigated laboratory findings before discharge, only at the presentation/baseline.

In contrast with our findings, other studies have reported a significant relationship between poor outcomes and decreased GFR levels at admission [16,23,25,26]. However, in our study, we observed a significant relationship between the outcome and creatinine and GFR levels two months after discharge. Low levels of creatinine and a normal GFR was associated with good outcomes. Few studies have specifically focused on the etiology, laboratory results, diagnosis, and clinical consequences of CrGN. There is limited information available on the disease patterns in Saudi Arabia and the Middle East. We performed this retrospective study to evaluate the clinic-pathological characteristics of CrGN in Saudi Arabia.

The limitation of our study is mainly related to the missing or non-reported histopathological results; patients with a clinical presentation of RPGN but without tissue biopsy were excluded, and the sample size of the study was small.

## Conclusions

Crescentic glomerulonephritis is a cause of acute renal failure. In our study, 12 out of 17 patients had poor outcomes, with lupus nephritis being the most common etiology. The factors associated with poor renal outcome included the presence of IFTA on biopsy, phosphate levels before discharge, creatinine levels before and after discharge, and GFR level after discharge. In our study, etiology was not related to the prognosis of CrGN. Early detection and treatment of CrGN are critical. Further studies from Saudi Arabia are required to identify the prognostic factors associated with CrGN.
